# Family physicians’ experiences with an innovative, community-based, hybrid model of in- person and virtual care: a mixed-methods study

**DOI:** 10.1186/s12913-023-09599-x

**Published:** 2023-06-03

**Authors:** Jonathan Fitzsimon, Kush Patel, Cayden Peixoto, Christopher Belanger

**Affiliations:** 1 Department of Family Medicine, University of Ottawa, 600 Peter Morand Crescent #201, Ottawa, ON K1G 5Z3 Canada; 2 Faculty of Medicine, University of Ottawa, Roger Guindon Hall, 451 Smyth Rd #2044, Ottawa, ON K1H 8M5 Canada; 3 Institut du Savoir Montfort, 713 Montréal Rd, Ottawa, ON K1K 0T2 Canada; 4 Department of Family Medicine, University of Ottawa, 600 Peter Morand Crescent #201, Ottawa, ON K1G 5Z3 Canada

**Keywords:** Hybrid care, Virtual care, Rural and remote, Primary care, Community paramedic

## Abstract

**Background:**

Rural, remote, and underserved communities have often struggled to provide adequate access to family physicians. To bridge this gap in Renfrew County, a large, rural region in Ontario, Canada, a community- based, hybrid care model was implemented, combining virtual care from family physicians and in-person care from community paramedics. Studies have demonstrated the clinical and cost effectiveness of this model but its acceptability to physicians has not been examined. This study investigates the experiences of participating family physicians.

**Methods:**

A mixed-methods study, combining physician questionnaire response data and qualitative thematic analysis of focus group interview data.

**Results:**

Data was collected from *n* = 17 survey respondents and *n* = 9 participants in two semi-structured focus groups (*n* = 4 and *n* = 5 respectively). Physicians reported high satisfaction, driven by skills development and patient gratitude, and felt empowered to reduce ED visits, care for unattached patients, and address simple medical needs. However, physicians found it difficult to provide continuous care and were sometimes unfamiliar with local healthcare resources.

**Conclusion:**

This study found that a hybrid model of in-person and virtual care from family physicians and community paramedics was associated with positive physician experiences in two main areas: clinical impacts, especially avoiding unnecessary ED visits, and physician satisfaction with the service. Potential improvements for this hybrid model were identified, and include better support for patients with complex needs, and more information about local health-system services. Our findings should be of interest to policymakers and administrators seeking to improve access to care through a hybrid model of in-person and virtual care.

**Supplementary Information:**

The online version contains supplementary material available at 10.1186/s12913-023-09599-x.

## Background

Robust primary care is crucial to a high-performing healthcare system [1, 2], and is associated with better health outcomes and lower healthcare cost [3]. However, providing robust primary care can be challenging in rural, remote and underserved communities due to travel barriers [4], and the often-inadequate supply of local family physicians [5, 6]. This regional lack of access to primary care can create localized healthcare inequities [7, 8], and can lead patients to forego care, or to resort to an emergency department (ED) for care that could otherwise have been dealt with in a community setting [9, 10]. Moreover, team based primary care may benefit patients with chronic disease by reducing the use of acute care [11, 12] and may reduce ED used [13].

To address these challenges, some jurisdictions are exploring “virtual care,” which involves providing healthcare through internet and telephone services, as a treatment modality for rural, remote, and underserviced areas [14, 15]. Virtual care use increased during the COVID-19 pandemic, especially during pandemic lockdowns [16, 17], and evidence is emerging that virtual care may be effective in strengthening access to primary care in rural and remote communities [18]. Findings show that more virtual care from family physicians during the COVID-19 pandemic did not result in more ED use [19]. However, especially in the context of the COVID-19 pandemic, there are many services that can only be delivered in person, such as COVID-19 nasal swab testing. This leaves open the possibility that virtual care delivery models may be more effective when integrated with in-person services.

This study examines an innovative, community based, hybrid model, the Virtual Triage and Assessment Center (VTAC), that combines virtual care from family physicians and in-person care from community paramedics. VTAC was established in Renfrew County, a rural county in Eastern Ontario, Canada, to provide all residents with access to COVID-19 assessment and testing and to prevent unnecessary visits to EDs. In keeping with the rapid changes to healthcare at the start of the pandemic, VTAC was developed and implemented at unprecedented speed, taking just 12 days from the initial planning phase to being operational [20–22]. VTAC provides access to a family physician for residents who have an urgent health concern but do not have a family physician or cannot access their regular primary care provider. Moreover, VTAC Family Physicians provided over 10,000 virtual appointments during the first six months of the COVID-19 pandemic [20]. In addition to care via telephone and video encounters, VTAC family physicians also have the option of requesting in-person assessment and follow up from community paramedics. This can be done directly through both written referrals for less urgent follow up and direct phone discussion with the VTAC paramedic dispatch desk, for more urgent (same day) or more clinically complex cases that still do not require an emergency 911 call. Community paramedics played a critical role in providing care during the pandemic, by providing COVID-19 testing at scheduled drive-thru sites, pop-up sites, and in-home for vulnerable, housebound patients. However, they can also perform physical examinations, monitor vital signs, and conduct a wide range of point of care testing, such as blood tests, glucose monitoring, urine analysis and rapid strep testing, at a patient's home or a fixed site or mobile assessment centre. The community paramedics have rapid access to VTAC physicians by phone and/or video, to help direct management planning, ED diversion and to refer for specialist consultation or to existing community healthcare resources, such as mental health supports and community palliative care. Overall, the close integration of family physicians and community paramedics reflects many attributes of team based primary care and increases access to primary care, particularly for the many residents who do not have a regular family physician or alternative primary care provider.

Previous research has demonstrated that VTAC may be an economical and effective way of delivering acute, episodic care in rural, remote, and underserved communities [20, 21, 23]. However, physician satisfaction and their experiences with delivering care through VTAC have yet to be investigated. This study employs quantitative and qualitative methods to explore perceptions and experiences of family physicians who provided care through VTAC during the first 18 months of the COVID-19 pandemic. Specifically, we enquired about the perceived benefits and limitations of VTAC, as well as preferences amongst family physicians who experienced both phone and video appointments. The purpose of this study is to examine physician perspectives of delivering care through VTAC during the COVID-19 pandemic. We also aim to provide recommendations for the development and improvement of VTAC and future hybrid models beyond the COVID-19 pandemic.

## Methods

### Study design

A mixed methods study design was employed to investigate the experiences of family physicians who provided care to patients through VTAC. This was done in the context of the quadruple aim framework for evaluating innovations and change in healthcare, looking for improved clinical outcomes, lower healthcare costs, enhanced patient experience and enhanced provider experience [24]. The clinical and economic impact of VTAC has been evaluated elsewhere [20, 21] and further work is underway to evaluate patient experience of VTAC. This study focuses on the physician experience of delivering care through VTAC and reports descriptive results from a study questionnaire, as well as results from a qualitative thematic analysis of focus group interview data. A qualitative research approach is appropriate for this study since we investigate an under-researched phenomenon and focus on individuals’ experiences and perceptions [25].

### Study recruitment

Study participation was open to any family physician who provided care through VTAC from March 27, 2020 (launch day) to August 11, 2021, able to understand and converse in English, and able to provide informed consent. All VTAC family physicians identified to meet the eligibility criteria (*n* = 22) were invited via e-mail to complete an online questionnaire (Additional file 1). The survey included an option for participants to indicate if they were interested in participating in an online focus group interview about their experiences providing care through VTAC and consent to be contacted further to schedule the study interview.

### Study sample and data collection

The online questionnaire (Appendix 1) collected demographic information and included Likert scale-type questions used to assess physician experience with VTAC (e.g., provider satisfaction). *N* = 17 participants completed the study survey. Of these, *n* = 9 responded that they would like to participate in an online semi-structured focus group interview regarding their experience delivering care through VTAC. Participants were not remunerated for their participation.

A total of two semi structured, focus group interviews (*n* = 4 and *n* = 5, respectively) were conducted using a set of predetermined questions and follow-up probes to explore provider perceptions of VTAC. These interviews were conducted online via Zoom, audio recorded, and transcribed using Zoom auto-generated transcription. These transcripts were then cross-referenced with the respective audio files to correct any inconsistencies and prevent transcription error. Due to the limited availability for focus group participation, we were only able to conduct two interviews. However, after reviews of the transcripts, we found that the same or similar themes appeared across both focus groups and concluded that the data was sufficiently saturated to report our results [26].

### Data analysis

This study conducted a conventional content analysis [27] led by two research team members, wherein focus-group transcripts were coded and categorized into recurring themes. Thematic analysis of study transcripts was performed using the qualitative analysis software NVivo version 1.5.2 [28]. The thematic analysis in this study uses as its conceptual framework, the widely accepted quadruple aim framework, a compass of optimizing health system performance [24]. This framework recognizes that physician burnout can contribute to worse patient care quality and that the main driver of physician satisfaction is being able to provide quality care to patients [29]. By analyzing the physician experience of delivering care to patients through VTAC, we were able to identify factors contributing to physician dissatisfaction that could be improved upon as well as determining if known contributory factors to physician satisfaction are present in the VTAC model.

We began analysis with thorough readings of the transcripts to develop an understanding of the data. Transcripts were reviewed individually before reflecting on interpretation of codes as a group. The two research team members then met to share key quotes from the transcripts, formulate meanings, and derive an initial coding scheme, which were sorted into broader themes. The coding scheme and themes were then shared among the other authors to increase rigor and develop a shared perception of content. We met to discuss and share their codes until a consistent level of consensus was established, adjusting the coding scheme based on shared meaning and coding frequency within the focus group transcripts [30]. Following coding of the transcripts, the authors met to ensure consistency in coding of quotes, as well as conduct thematic analysis to identify and define themes in the data [31]. In addition, the study follows the Standards for Reporting Qualitative Research (SPQR) reporting guidelines [32].

## Results

The demographics of the *n* = 17 survey participants are shown in Table 1. There was adequate representation from different age groups, levels of experience, genders, and types of practice among the participants.

**Table 1:** Demographic information of study participants (*n* = 17)

Demographic Information	Total n (%)
**Age**	
25–34	7 (41)
35–44	5 (29)
44–54	4 (24)
> 55	1 (6)
**Gender**	
Female	11 (65)
Male	6 (35)
**Type of practice**	
Private	3 (18)
Group	8 (47)
Hospital based	2 (12)
Locum tenens	4 (24)
**Location of practice**	
Renfrew County	12 (71)
Champlain region	3 (18)
Elsewhere in Ontario	1 (6)
No base practice	1 (6)
**Years as qualified family physician**	
0–5	8 (47)
6–10	3 (18)
11–15	3 (18)
16–20	2 (12)
21 +	1 (6)
**Number of VTAC appointments via video**	
0	12 (71)
1–5	4 (24)
6–10	1 (6)
Total	17

**Fig. 1:**
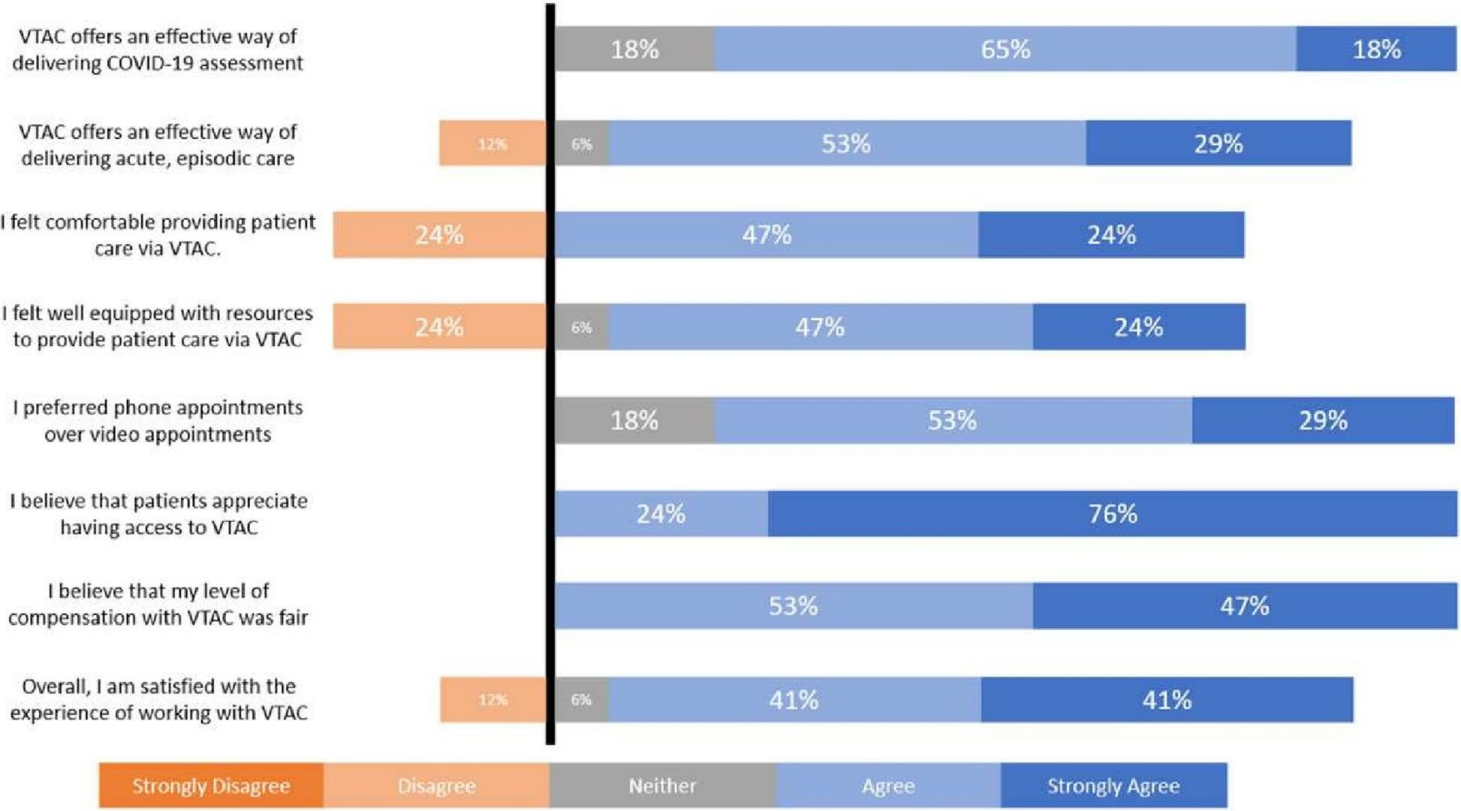
Participants’ perceptions of the hybrid care model from survey data, *n* = 17/22. *Values may not always sum to 100% due to rounding

**Fig. 2:**
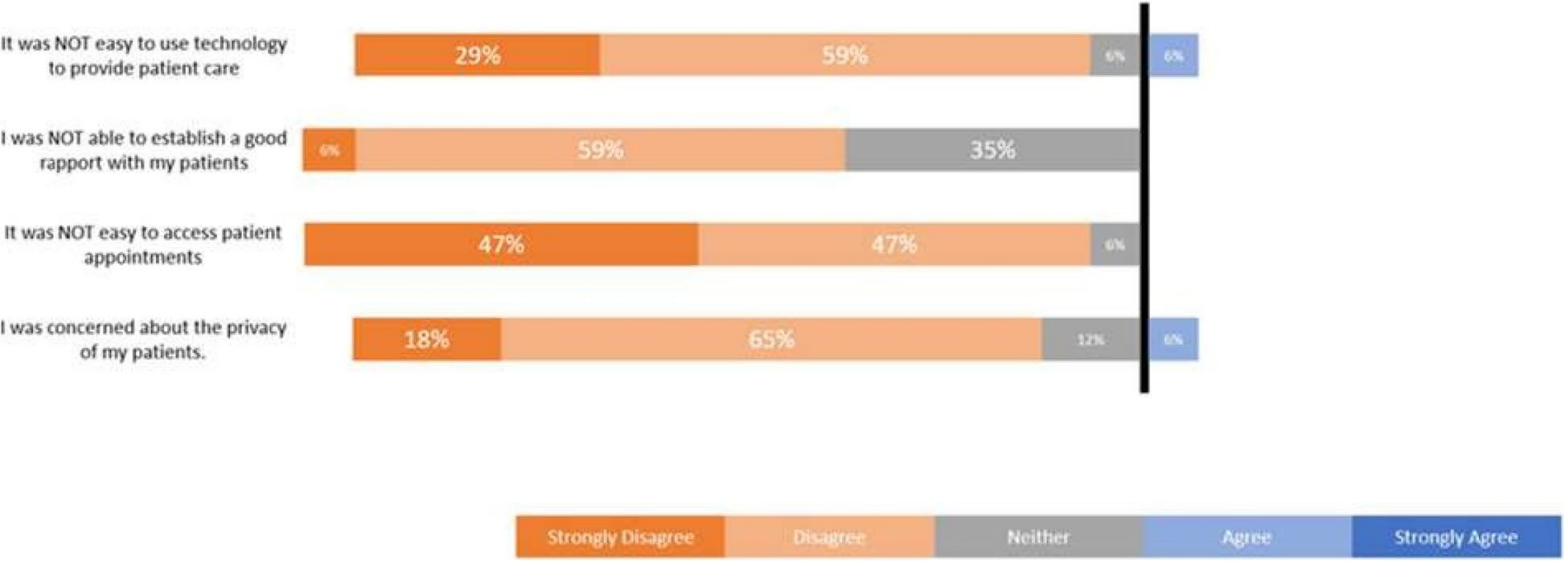
Participants’ perceptions of the hybrid care model from survey data, *n* = 17/22 (Continued). *Values may not always sum to 100% due to rounding

The Likert-scale data from the survey is illustrated in Figs. 1 and 2. Overall, most participants were very satisfied working for VTAC. They believed it was an effective way of delivering both COVID-19 assessment and other acute, episodic care. Most participants reported no concerns regarding patient privacy when using VTAC and found it easy to provide care using technology. All participants accessed appointments easily, preferred phone appointments, received fair compensation, established good rapport with patients, and believed that their patients appreciated access to VTAC.

A sub-analysis of the survey examining local versus non-local physicians revealed that out of the 17 participants, 15 were based in or close to Renfrew County, while the remaining 2 were practicing in other parts of Ontario. Interestingly, the non-local physicians expressed no negative perceptions of the model. It was the local physicians who provided critical feedback on the survey. The focus group consisted of 1 non-local physician and 8 local physicians.

The focus group data helped to further explore the strengths and weaknesses of VTAC. Three main concepts were identified: physician impact, physician satisfaction, and physician challenges. Our analysis deduced three themes for physician impact: triaging ER visits, reaching unattached patients, and improving patient care; two themes for physician satisfaction: personal fulfilment and professional development; and three themes for physician challenges: delivering non-continuous care, navigating community resources, and practicing via videoconference. We describe these themes in the following sections and illustrate them with anonymized quotations from focus-group participants.

### Physician impact

Participants frequently commented on how VTAC was able to help them positively change the lives of Renfrew County residents, especially those without family physicians, by providing them with a service that addressed their urgent health needs. Participants described how VTAC enabled them to save healthcare resources by *triaging ER visits* which decreased ER volumes: “we are getting back to pre- pandemic volumes in ER, but not nearly as high, and I think that is definitely due to VTAC” (P2). Further, some participants defined identifying when emergency care was required, as just as much of a success as being able to deal with an issue without resorting to additional care: “I feel good when I save ER visits, but when I would want them to go to the ER, I feel like that is a success as well” (P7).

VTAC’s hybrid combination of virtual and in-person care was seen as an enabler for *reaching unattached patients*. Many VTAC patients were seniors, often without a family physician and some with mobility restrictions. Participants in the discussion agree that the integration of Community paramedics into the VTAC has been a successful part of the program. It was “particularly helpful for linking paramedic care to the Community, especially for patients who are unable to travel and require complex care” (P4). Another physician recounted a specific instance when the Community paramedics used technology to enhance patient care, saying "I remember a visit where the paramedics were in the home, I was on the phone, they had a tablet with a camera so they were able to do sort of real-time video and pictures of this wound that the patient wouldn't have been able to manage…it worked really well." (P5). The option for VTAC physicians to augment their virtual care with in-person support from a paramedic was seen as an important part of this hybrid service, “I think that’s actually one of the most successful parts of VTAC” (P4).

Several participants expressed “that a service like VTAC needs to stay around” because the care it offered to unattached patients would not be possible in “the Ottawa valley without VTAC at this point unless they get more family doctors” (P8). There was also discussion about adopting this model of care for other underserviced areas to supplement their primary care.

Other participants described their experiences of *improving patient care* by interim management of chronic diseases and preventative care, and referrals to facilitate specialized care and follow up: “we are putting patients in touch with mammograms, FIT tests, referrals to gynecology” (P2). This also included review and renewal of prescriptions for patients. During virtual appointments, “there were a lot of prescription renewals that spilled into VTAC,” and they were successfully addressed (P2). In summary, nearly all participants described ways VTAC enabled them to increase their positive impacts on both patients and the healthcare system.

### Physician satisfaction

Providing care through VTAC cultivated the participants’ clinical skills and patient feedback boosted their morale. Most participants touched on the *personal fulfillment* they experienced from giving patient centered care: “If I can help the patient in a way that they were hoping for, or that I think is appropriate, I think that seems to be the case for the most part” (P8). The gratitude of patients motivated participants at work and helped them see the value of a service like VTAC for underserved communities.

VTAC also served as a *professional development* opportunity for participants to hone their skills which they could apply to “other areas of practice” (P3). Some participants explained that VTAC made them “a lot better at taking a really good history” due to limitations of the virtual encounter (P3). One participant that was in an urban center noted that they gained insight into treating patients from rural areas.

### Physician challenges

Participants experienced some difficulties caring for patients with complicated issues, knowing about the full range of available local services in the patient’s community, and with video appointments. If a patient requires follow up after their first VTAC appointment, it is not possible to book with specific providers which leads to *delivering non-continuous care.* This can be problematic for patients with multiple or complex issues requiring regular follow up: “In a 10-min period you can’t cover everything or be as detailed” and patients are “adamant about talking to the same doctor” (P7).

Participants also discussed how prescribing opioids and other narcotics posed particular difficulties.

Participants that were located outside of Renfrew County encountered the added challenge of *navigating community resources*: “Sometimes I feel lost with patients, because I don't know what resources they have access to” (P9). Other participants had a hard time ordering and following up on medical investigations, especially when it involved using provincial computer systems outside of VTAC’s control: “it takes me forever to trace my own investigations… that drives me crazy and it's very time consuming” (P1). Most physicians did not experience these challenges. However, when these issues did arise, they could compound the concerns about non-continuous care described above; importantly, these problems had been recognized internally just prior to the focus groups, and swift action was initiated to remediate them.

Participants found that *practicing *via* videoconference* was a challenge for parts of Renfrew County, due to inequitable high speed internet access. One participant noted that it could be difficult to get a good internet connection with a patient, and “even if you can get it, it’s so broken or so pixelated it’s useless” (P1). This issue is compounded by the fact that “most of the patients or at least 50% that we see are older and not necessarily tech savvy” (P2).

## Discussion

### Main findings

In this mixed methods study, we examined physician perspectives of delivering care through VTAC during the COVID-19 pandemic. Our questionnaire identified that COVID-19 assessments, acute episodic care, and overall physician satisfaction were strengths of VTAC, while the challenges of supporting patients with complex care needs with continuous care were limitations. Focus-group interviews with VTAC family physicians were conducted to further explore these ideas, and our qualitative analysis found three main saturated themes within the data: physician impact, physician satisfaction, and physician challenges.

Our results demonstrate that VTAC was associated with positive physician experiences in two major areas: (1) the clinical impacts family physicians were able to deliver, and (2) the satisfaction they derived from using the service.

We also identified some areas for improvement, where physicians were less satisfied and felt that the program had the potential to provide even more benefits. In particular, physicians felt that their experience would have been enhanced with firmer guidance on controlled substance prescribing, better information about local health-system services, streamlined registration for external IT systems, and with more support to enable them to better care for patients with complex care needs. Through the research process, these issues have been raised with VTAC program management as quality- improvement opportunities, and have already resulted in improvements, such as enhanced protocols for prescribing controlled substances and improved workflows for safely prescribing long term medications [33].

### Recommendations

Based on our findings, we make two recommendations for those considering a local program of hybrid in-person and virtual care such as the VTAC model. First, since participating providers may be serving clients outside their normal practice geography, we recommend creating a centralized provider-friendly knowledge base of region-wide health-system resources, which would serve as a reference for providers. This could be as simple as a shared document containing addresses and phone numbers for different clinics and services, or as complicated as a "wiki" or database, as long as it provides a centralized and used-friendly store of local knowledge to assist remote providers.

Second, we recommend having well-defined goals, regular opportunities to receive and act on feedback from providers, and a strategic development process that includes a deep knowledge of the target population, and cooperation and coordination with other healthcare providers and services in the regional healthcare system. VTAC is designed to provide acute, episodic care for patients who have urgent needs, but do not have a family physician or cannot access their usual primary care provider. Participants in our study were very satisfied with the level of care they could deliver to these patients in this context. VTAC is not designed to provide ongoing comprehensive care for patients with complex care needs, and participants in our study were less satisfied with the level of care they could provide to these patients.

Renfrew County is working to expand VTAC's "sister program", Integrated Virtual Care (IVC), which provides attachment to a family physician and team-based, comprehensive primary care. VTAC is leveraged to identify patients suitable for admission to the IVC program. Innovative programs such as IVC, that provide permanent attachment to a family physician and comprehensive, team-based care are part of the longer-term solution to the problems of insufficient access to primary care. VTAC can act as a safety net for patients without access to a family physician, until such a time as they have access to comprehensive primary care.

### Limitations of the study

This study has several limitations that should be considered when interpreting its results. This study focused on the experience of physicians delivering care through VTAC and does not include an evaluation of the experience of paramedics. We interviewed about half of VTAC's participating physicians, but they may not be a representative sample of physicians overall. In addition, all participants worked in the province of Ontario, Canada, and results may not generalize to other countries or cultures. Lastly, focus groups were conducted remotely, so it is unknown what the impact of doing them in person might have been.

### Future directions

Future work could extend this study in several directions. First, as this study's findings are translated into ongoing quality improvement initiatives, future work could help to understand physicians' perceptions of these initiatives and identify further opportunities for improvement. Another promising avenue would be to investigate the major gap physicians identified, the level of care they are able to offer to patients with complex needs. A study of these patients and their perceptions and needs could help to identify ways to better serve them through existing or new local initiatives, including new opportunities offered through virtual care. Further work is ongoing to garner a more fulsome picture of other providers’ experience within VTAC, particularly that of paramedics, as well as to evaluate the experience of patients accessing care through VTAC.

## Conclusion

Overall, VTAC’s family physicians felt empowered to reduce ED visits, care for unattached patients, and address acute, episodic medical needs. Physician satisfaction was driven by patient gratitude and skills development. However, physicians found it difficult to provide continuous care, and physicians from different locations were not always fully aware of all local community resources available to patients. Our findings suggest that a community based, hybrid model of in-person and virtual care can enhance the experience of family physicians providing care to patients who do not have a family physician or cannot access their regular primary care provider.

## Supplementary Information

**Additional file 1.** VTAC Physician Experience Survey.

**Additional file 2:** **Table S1.** Preliminary Findings: Data Structure and Major Themes. **Table S2.** Recommendations and improvements.

## Data Availability

Examples of raw data from the study, i.e., focus group quotes, can be found in the supplementary materials (Tables S1 and S2). The full dataset used and analyzed in the current study are available from the corresponding author on reasonable request.
