# Supplementary material for: Family physicians’ experiences with an innovative, community-based, hybrid model of in- person and virtual care: a mixed-methods study
Source: BMC Health Serv Res. 2023 Jun 3;23:573. doi: 10.1186/s12913-023-09599-x (PMC10238231; doi:10.1186/s12913-023-09599-x)
Supplement: Supplementary file 1 — Additional file 1. VTAC Physician Experience Survey. [file 12913_2023_9599_MOESM1_ESM.pdf]

**Additional file 1 – VTAC Physician Experience Survey**

**1.** What is your age?

- 18-24
- 25-34
- 35-44
- 45-54
- Over 55

2. What is your gender?
   - Female
   - Male
   - Non-binary

3. What it you year of graduation from medical school?
   - List years 1961 to 2021

4. What is your type of practice?
   - Private
   - Group
   - Hospital-based
   - Locum Tenens

5. What is your location of practice?
   - Renfrew County
   - Champlain Region
   - Elsewhere in Ontario
   - No Base Practice

6. How many years have you been in your current role?
   - 0-5
   - 6-10
   - 11-15
   - 16-20
   - 21-25
   - 26-30
   - Over 31
7. How many days per week do you work in your practice?
   - 1
   - 2
   - 3
   - 4
   - 5
   - 6
   - 7

8. What shift do you prefer?
    - Morning
    - Afternoon
    - Evening
9. What percentage of your VTAC appointments were via phone verses video?
    - 0-20
    - 21-40
    - 41-60
    - 61-80
    - 81-100

Likert scale for the following statements:
1 - Strongly disagree
2- Disagree
3 – Neither agree or disagree
4 – Agree
5 – Strongly Agree

10. VTAC offers an effective way of delivering COVID-19 assessment.

11. VTAC offers an effective way of delivering acute, episodic care.

12. I felt comfortable providing patient care via VTAC.

13. I felt well equipped with resources to provide patient care via VTAC.

14. It was NOT easy to use technology to provide patient care.

15. I was NOT able to establish a good rapport with my patients.

16. It was NOT easy to access patient appointments.

17. I preferred phone appointments over video appointments.

18. I was concerned about the privacy of my patients.

19. I believe that patients appreciate having access to VTAC.

20. Overall, I am satisfied with the experience of working with VTAC.

21. Do you give the research team permission to contact you to participate in the focus group?
    - Yes
      First-name and last name: ______________________________________

E-mail address: ______________________________________________
Phone number: ______________________________________________

- No
  Voluntary participation: We recognize that you have invested time in our study and you are free to participate or not in this activity of the study. Regardless of your decision we will use the data you already provided and use it as planned unless you have asked for them to be removed.

If you would like to withdraw your data, please contact Kush Patel (VTAC Research Assistant) at kupatel@ohri.ca.
