# Supplementary material for: Family physicians’ experiences with an innovative, community-based, hybrid model of in- person and virtual care: a mixed-methods study
Source: BMC Health Serv Res. 2023 Jun 3;23:573. doi: 10.1186/s12913-023-09599-x (PMC10238231; doi:10.1186/s12913-023-09599-x)
Supplement: Supplementary file 2 — Additional file 2: Table S1. Preliminary Findings: Data Structure and Major Themes. Table S2. Recommendations and improvements. [file 12913_2023_9599_MOESM2_ESM.pdf]

SUPPLEMENTARY MATERIAL

Table S1. Preliminary Findings: Data Structure and Major Themes.

	Themes	Description	Representative Quotations	1st-Order Concepts
**Physician Impact**	Triaging ER Visits	Reducing unnecessary and validating appropriate ER visits to benefit both the patient and the health care system	• P02: We are getting back to pre-pandemic volumes in ER, but not nearly as high, and I think that is definitely due to VTAC.<br>• P05: Success is when you can tell somebody exactly what the issue is, and they don't have to go to ER. You've saved them a trip and you've saved the healthcare system some time and money.<br>• P06: They say, "thank you for telling me I need to go to the ER." Sometimes that's a reward. It's validating the severity of their medical concern.<br>• P07: I feel good when I save ER visits, but when I would want them to go to the ER, I feel like that is a success as well.<br>• P09: I think VTAC is basically going to fill the role for non-urgent emergency room visits. Pre-pandemic, a lot of the patients with non-urgent issues would go to the ER.	• Lower ER volume<br>• Less ER visits for non-urgent issues<br>• More ER visits for urgent issues
	Reaching Unattached Patients	Reaching unattached and vulnerable patients and providing them with access to primary care	• P07: If they lose their family doctor when they're 80 plus years old, then VTAC will fill a great void for those patients. I have quite a few patients asking if it's going to continue. A lot of the elderly people are very vulnerable and have to go sit in ED for hours on end waiting for prescription.<br>• P08: I don't even know how the Ottawa valley would do without VTAC at this point unless they get more family doctors. I really think that a service like this needs to stay around, especially for the patients who don't have a family doctor. For most of my patients, it's not even a COVID question. It's usually more family medicine questions.	• Lack of and retiring family physicians leaves many unattached patients<br>• Essential service to access primary care, especially for older population<br>• Other rural communities can benefit from this model

			• P09: More people are going to hear about it, and hopefully we can deliver this access to care to not just the Ottawa Valley people but northern Ontario communities as well.	
	Improving Patient Care	Helping patients by providing medical advice, prescriptions, referrals, and chronic disease management that they otherwise would have gone without.	• P02: There were a lot of prescription renewals that spilled into VTAC… we're seeing less and less of that in ED, so that's an example to illustrate how VTAC is really helping.<br><br>• P07: There's a lot of pregnant women who don't have a family doctor and don't have an OB. So, you can initiate the bloodwork, the initial ultrasound, the first trimester screen… and then refer to OB. That's a great asset of VTAC.<br><br>• P09: Mental health patients mentioned that they had trouble seeking care simply because they didn't want to be face to face with providers, and they found it a lot easier to speak to me over the phone where they have that barrier.	• Manage prescriptions and simple medical issues effectively<br><br>• Initiate screening and monitoring tests<br><br>• Prevent disease progression
**Physician Satisfaction**	Personal Fulfilment	Addressing patient concerns is rewarding for physicians	• P02: But I think that's sort of the thing that kind of keeps me going because, like obviously there's sort of that safety and privacy issues that we've all mentioned but for me having patients actually appreciating the service and being thankful that were there, I think that's really helpful for me to continue to provide the service that I do.	• Patient satisfaction motivates physicians<br><br>• Valuable patient service and physician experience

			• P08: I think for me successes, if I can help the patient in a way that they were hoping for, or that I think is appropriate, I think that seems to be the case for the most part.<br><br>• P08: I thought that most of these visits would just be sort of prescription renewals but I actually felt that the service that I was able to provide when quite beyond that and so I do feel like you know there's a need I'm able to help so it's been very rewarding in that sense and has made me really realize how important. It is for the patients that are being served, and I really hope it continues, because I don't know what some of them would do without it.	
	Professional Development	Improving history-taking and strategic thinking skills through exposure to different cases	• P03: I have gotten a lot better at you know, taking a really good history because we're limited we don't have the physical exam we often don't have you know, a patient profile with a list of their medication, so I think in that sense. You know, it has made me a stronger history taker. Which has helped me in other areas of my practice.<br><br>• P09: It opened up a lot more interesting cases for me to see because when I work basically in an urban area where we see a lot of sin cases but seeing a lot of rural cases as well, was also interesting so kind of diversify my exposure.	• Virtual limitations foster comprehensive histories and foresight<br><br>• Greater exposure to rural cases
**Physician Challenges**	Delivering Discontinuous Care	VTAC patients may see a different physician each visit, which can be disorienting for patients and make it difficult for physicians to care for patients with complex conditions and	• P07: Sometimes the cases are so complex that in a 10-minute period you can't cover everything or be as detailed. So, you need them to call back for another appointment to address their concerns or send them to ER where many things can be done at once…labs or diagnostic imaging and have follow up that same day.<br><br>• P07: I find that older patients want to talk to you again. Trying to explain to them that that's not the way VTAC works. I've had a few in the last month that have been quite adamant about just talking to the same doctor. You kind of feel bad for them for having a family doctor for 40 years and now they're going through virtual medicine and talking to a completely different doctor every time.	• Lack of time and resources in virtual setting<br><br>• Limited options for opioid prescriptions<br><br>Patient preference for same physician

	opioid prescriptions.	P08: I think opioid prescriptions are a challenge. I understand our policy is to not refill opioid prescriptions. People on chronic opioids that have been initiated by previous family doctor and the doctor retired, so only option is ER. I feel for these patients because what are they supposed to do.	
Navigating Community Resources	Lacking familiarity with community resources in areas other than location of practice	• P01: I don't know the best place to do [a specific test], or I don't know what they actually have. I live in the bottom part of the valley, so I know how I would coordinate all of that down here, but I don't know up there.<br>P09: Unless you're from the area the patient is calling from, it's hard to know what kind of resources are available... Sometimes I feel lost with patients, because I don't know what resources they have access to.	• Coordination of medical investigations<br>• Knowledge of local community services
Practicing via Videoconference	It can be challenging to practice medicine via internet teleconferencing due to inequitable rural internet access and technical issues.	• P01: I would use video, but huge part of the valley just doesn't have the connectivity. Even if you can get it, it's so broken or so pixelated it's useless.<br>• P02: Most of the patients or at least 50% that we see are older, not necessarily tech savvy.<br>• P07: They’re either too closer too far away or just the resolution of their devices is not very clear, and so it makes it difficult. Sometimes we just ended up asking them to send pictures, as opposed to using the video.	• Lack of internet connectivity<br>• Older population and technology proficiency<br>• Good quality picture is better than low quality video

Table S2. Recommendations and improvements.

Themes	Description	Representative Quotations	1st-Order Concepts
Adding In-person Assessments	To sustain continuous and complex follow-up care of patients, VTAC necessitates an in-person component whether it be a mobile team, rotating physician schedule or allied healthcare professionals	• P03: I like this idea of a mobile team or rotation of physicians, maybe we did like a week here a week there. Ideally, we would want long term physicians that are going to stay in the area, but more realistically a rotation schedule, where you don't have to commit to being the long-term physician in that area for years.<br>• P04: I was hoping that in the future, virtual care would transfer to in person if the patient required physical exam. There's a nurse practitioner in the clinic that is designated for VTAC. So rather than shifting the care into the emergency department, we get them to come in person assessment.<br>• P05: I remember a visit for the paramedics, I was on the phone, they had a tablet with a camera. I'm looking at the pictures on my cell phone and talking on my other phone and that worked really well. I wonder if a mobile team, maybe independent from the paramedic service would be feasible add on. VTAC's got a great role to play, but I just struggle with the idea that sometimes you just need to see people. How can it be sustainable without any component of physical exam and meet a real standard of care.	• Offer in-person visit option to patients<br>• Mobile team with videoconferencing technology<br>• Clinic assessments by physicians or nurse practitioners
Updating and Communicating Policies	Policies should be reconsidered when they prevent patient centered care like prescribing opioid and all team members should be made aware of amendments	• P01: I would like to think that all been thought out and maybe I don't know but from the rest of the blank looks, I suspect, nobody else knows either so that could just be some good communication to help fix that.<br>• P08: I think to strictly not do opiates at all. I don't know if that's really the best option, and maybe that could be revisited to have an intermediate solution. I've called	• Revisit opioid policy<br>• Clear internal communication

		pharmacies, to confirm someone's prescription. You can ask the pharmacy what exactly they are on, how often are they getting it, just to make sure that it's legit and they're not kind of seeking for something. That could maybe be an option.<br>• P09: We did actually adjust that policy. We agreed that we're not going to prescribe any hard narcotics like hydromorphone or fentanyl. When prescribing a weaker ones and benzodiazepines, if they were on chronically we're going to prescribe it for them.	
Implementing a Community Resource Tool	A list of resources such as labs and allied health partners should be complied to assist physicians that are not familiar with Renfrew County	• P01: And I think that could easily be fixed by actually having a better sort of resource thing on what's available in each community to do.<br>• P08: It would be awesome if we had some people who are knowledgeable, if they were able to put together something for resources and those could be available in the documents section. That would be very helpful.	• Staff familiar with local services<br>• Document with resources

Standards for Reporting Qualitative Research (SRQR)*

http://www.equator-network.org/reporting-guidelines/srqr/

Page/line no(s).

Title and abstract

**Title** - Concise description of the nature and topic of the study Identifying the study as qualitative or indicating the approach (e.g., ethnography, grounded theory) or data collection methods (e.g., interview, focus group) is recommended	Title page, p.1
**Abstract** - Summary of key elements of the study using the abstract format of the intended publication; typically includes background, purpose, methods, results, and conclusions	Abstract, p.2

Introduction

**Problem formulation** - Description and significance of the problem/phenomenon studied; review of relevant theory and empirical work; problem statement	Introduction, p.4-5
**Purpose or research questio**n - Purpose of the study and specific objectives or questions	Introduction, p.5

Methods

**Qualitative approach and research paradigm** - Qualitative approach (e.g., ethnography, grounded theory, case study, phenomenology, narrative research) and guiding theory if appropriate; identifying the research paradigm (e.g., postpositivist, constructivist/ interpretivist) is also recommended; rationale**	Study design, p.5-6;<br>Data analysis, p.6-7
Researcher characteristics and reflexivity - Researchers' characteristics that may influence the research, including personal attributes, qualifications/experience, relationship with participants, assumptions, and/or presuppositions; potential or actual interaction between researchers' characteristics and the research questions, approach, methods, results, and/or transferability	Data analysis, p.6-7
**Context** - Setting/site and salient contextual factors; rationale**	Introduction, p.4-5
**Sampling strategy** - How and why research participants, documents, or events were selected; criteria for deciding when no further sampling was necessary (e.g., sampling saturation); rationale**	Study recruitment, p.5-6;<br>Study Sample and Data Collection, p.6
**Ethical issues pertaining to human subjects** - Documentation of approval by an appropriate ethics review board and participant consent, or explanation for lack thereof; other confidentiality and data security issues	Ethical Considerations, p.7

**Data collection methods** - Types of data collected; details of data collection procedures including (as appropriate) start and stop dates of data collection and analysis, iterative process, triangulation of sources/methods, and modification of procedures in response to evolving study findings; rationale**	Study Sample and Data Collection, p.6
**Data collection instruments and technologies** - Description of instruments (e.g., interview guides, questionnaires) and devices (e.g., audio recorders) used for data collection; if/how the instrument(s) changed over the course of the study	Study Sample and Data Collection, p.6
**Units of study** - Number and relevant characteristics of participants, documents, or events included in the study; level of participation (could be reported in results)	Results, Table 1, p.7-8
**Data processing** - Methods for processing data prior to and during analysis, including transcription, data entry, data management and security, verification of data integrity, data coding, and anonymization/de-identification of excerpts	Study Sample and Data Collection, p.6; Data analysis, p.6-7
**Data analysis** - Process by which inferences, themes, etc., were identified and developed, including the researchers involved in data analysis; usually references a specific paradigm or approach; rationale**	Data analysis, p.6-7
**Techniques to enhance trustworthiness** - Techniques to enhance trustworthiness and credibility of data analysis (e.g., member checking, audit trail, triangulation); rationale**	Data analysis, p.6-7

Results/findings

**Synthesis and interpretation** - Main findings (e.g., interpretations, inferences, and themes); might include development of a theory or model, or integration with prior research or theory	Results, p.7-12
**Links to empirical data** - Evidence (e.g., quotes, field notes, text excerpts, photographs) to substantiate analytic findings	Results, p.7-12; Supplementary Materials - Data Structure, Table S1; Supplementary Materials – Recommendations and Improvements, Table S2

Discussion

**Integration with prior work, implications, transferability, and contribution(s) to the field -** Short summary of main findings; explanation of how findings and conclusions connect to, support, elaborate on, or challenge conclusions of earlier scholarship; discussion of scope of application/generalizability; identification of unique contribution(s) to scholarship in a discipline or field	Discussion: main findings, p.11-13
**Limitations** - Trustworthiness and limitations of findings	Limitations, p.13

Other

**Conflicts of interest** - Potential sources of influence or perceived influence on study conduct and conclusions; how these were managed	Declaration of Competing Interests, p.1
**Funding** - Sources of funding and other support; role of funders in data collection, interpretation, and reporting	Sources of Funding, p.1; Declaration of Competing Interests, p.1

*The authors created the SRQR by searching the literature to identify guidelines, reporting standards, and critical appraisal criteria for qualitative research; reviewing the reference lists of retrieved sources; and contacting experts to gain feedback. The SRQR aims to improve the transparency of all aspects of qualitative research by providing clear standards for reporting qualitative research.

**The rationale should briefly discuss the justification for choosing that theory, approach, method, or technique rather than other options available, the assumptions and limitations implicit in those choices, and how those choices influence study conclusions and transferability. As appropriate, the rationale for several items might be discussed together.

Reference:

O'Brien BC, Harris IB, Beckman TJ, Reed DA, Cook DA. Standards for reporting qualitative research: a synthesis of recommendations. Acad Med. 2014 Sep;89(9):1245-51.
doi: 10.1097/ACM.0000000000000388. PMID: 24979285.
